# Retention and sustainability of community-based health volunteers' activities: A qualitative study in rural Northern Ghana

**DOI:** 10.1371/journal.pone.0174002

**Published:** 2017-03-15

**Authors:** Samuel Chatio, Patricia Akweongo

**Affiliations:** 1 Navrongo Health Research Centre, Navrongo, Ghana; 2 University of Ghana, School of Public Health, Accra, Ghana; Foundation for Medical Research, INDIA

## Abstract

**Background:**

The shortage of formal health workers has led to the utilization of Community-Based Health Volunteers (CBHV) to provide health care services to people especially in rural and neglected communities. Community-based health volunteers have been effective partners in health care delivery at the community level for many years. The challenge is how to retain these volunteers and also sustain their activities. This study explored factors affecting retention and sustainability of community-based health volunteers’ activities in a rural setting in Northern Ghana.

**Methods:**

This was a qualitative study comprising thirty-two in-depth interviews (IDIs) with health volunteers and health workers in-charge of health volunteers’ activities. Purposive sampling technique was used to select study participants for the interviews. The interviews were transcribed and coded into themes using Nvivo 10 software. The thematic analysis framework was used to analyze the data.

**Results:**

Study participants reported that the desire to help community members, prestige and recognition as doctors in community mainly motivated them to work as health volunteers. Lack of incentives and logistical supplies such as raincoats, torch lights, wellington boots and transportation in the form of bicycles to facilitate the movement of health volunteers affected the work. They suggested that lack of these things discouraged them from working as health volunteers. Most of the dropout volunteers said lack of support and respect from community members made them to stop working as health volunteers. They recommended that community support, incentives and logistical supplies such as raincoats, torch light, wellington boots, bicycles, awards to hard working volunteers are mechanisms that can help retain community-based health volunteers and also sustain their activities.

**Conclusion:**

Providing means of transport and non-monetary incentives would help to retain community-based health volunteers and also sustain their activities at the community level.

## Introduction

The shortage of formal health workers to provide health services has made it necessary for health managers to use Community-Based Health Volunteers (CBHV) to provide health care services to people especially in rural or neglected communities [1]. These health volunteers are selected by their own community members and trained to support professional health workers to provide basic health care services to people in communities that lack access to adequate health care services. This is particularly the case in setting with a shortage of professional health workers [2, 3].

Community health volunteers have been effective partners and have played an important role in helping to promote healthy behaviors, prevent diseases and case management of sick children [2]. It is demonstrated that CBHVs’ involvement in health care services can help reduce morbidity, mortality and fertility. [4,5,6]. The activities of health volunteers have contributed significantly to increase parents and family caregivers’ knowledge in managing fever and diarrhea in children less than five years [7]. Health volunteers also offer home based counseling to mothers and the treatment of uncomplicated childhood illnesses [8].

Despite the important role played by CBHVs in health service delivery at the community level over the years, the major challenges include how to retain health volunteers and also sustain their activities. Previous studies have shown that effective training, supervision, appreciation by community members and family support are all factors influencing retention of health volunteers. [9,10,11]. In addition, community-directed health interventions mostly fail because of lack of funds at the local level to motivate CBHVs engaged in health intervention activities [12]. Although health volunteerism has been described as community members offering free services to their own people at the community level, there is lack of consensus on whether CBHVs should be given allowance or non-monetary incentives and logistics to help sustain their activities. The level of community involvement in programme designing and implementation, the selection processes of CBHVs and the workload also greatly influence retention of health volunteers and sustainability of their activities at the community level [12].

In line with the Primary Health Care declaration, Ghana tried several strategies immediately after independence in 1957 to make health care services available, accessible and affordable to the people especially those in rural communities. The plan adopted was to promote community-based health care through community involvement and volunteerism concept in the late 1970s [13]. The initiative was the government’s plan to make health care services available at the doorsteps of people through community participation, ownership and volunteerism [13]. During early 1990s, more than 70% of all Ghanaians lived more than eight kilometers from the nearest healthcare provider and rural infant mortality rates were 50% higher than the urban rates [13]. Therefore, improving access to healthcare delivery in rural communities became the primary focus of health sector reforms in the 1990s. The Ghana’s Ministry of Health introduced the health volunteer programme in the late 1970s to provide community-based primary health care. Health volunteers were selected, trained and allowed to practice with minimal supervision at the community level [4,14]. Despite many years of involvement of health volunteers in health programmes in Ghana, the main challenge on the volunteer concept has been how to retain them. Previous studies have reported 31% to 44% dropout rate few years after the start of the volunteers’ program [15, 16]. Therefore, this study explored factors affecting retention of health volunteers and sustainability of their activities in the Kassena-Nankana Districts of Northern Ghana.

## Methodology

### Ethical approval

Ethics approval for the study was obtained from the Ghana Health Service ethics review committee. Approval was also sought at the Upper East Regional Health directorate and at the district health management offices in the two Kassena-nankana districts before data collection started. Written informed consent was obtained from health workers and health volunteers before they were interviewed. Study participants were told about the purpose of the study, how they were selected to take part in the study, their right to refuse being interviewed, their right to withdraw in the process of the interview and confidentiality of the information provided.

### Study area

The study was conducted in the Kassena-Nankana East district (KNED) and Kassena-Nankana West District (KNWD). The Districts are located in the north-eastern part of Ghana, and borders with Burkina Faso in the north. The two districts cover an area of 1,675 square kilometres of Sahelian savannah with a population of about 153,000 under surveillance by the Navrongo Health Demographic Surveillance System (NHDSS) [17]. The main languages spoken in the two districts are Kasem and Nankani. The population is predominantly rural with subsistence farming as the mainstay of the districts’ economy with the main crops being millet, sorghum, rice, maize and groundnuts. People live in multi-family compounds which form the basis of an address system used by the NHDSS. Majority of the people live in rural and sparsely scattered settlements. With the dispersed settlement pattern in the districts, health care service delivering is often very difficult.

The districts have 1 hospital, 8 health centers/clinics, two private clinics and 28 Community-based Health Planning and Services (CHPS) compounds located in various communities providing health care services to people [18, 19]. There are CBHVs in all the communities in the two districts supporting professional health workers to provide basic health care services to people.

### Study population/Sampling technique

All community-based health volunteers in the two districts qualified to take part in this study. Health volunteers who have worked for long (five years and above) and dropout volunteers were also identified and interviewed. Health workers in-charge of volunteer activities at the sub-districts level were also interviewed. The health professionals shared their experiences and views on how to retain CBHVs and also sustain their activities in the study area. The long serving health volunteers on the other hand shared their experiences on the factors that motivated them to work for long periods while the dropout volunteers explained the reasons why they left the voluntary work.

The two districts have been divided into sub-districts and each sub-district has a number of communities with two health volunteers working in each community. KNED has a total of six sub-districts, one hundred and fifteen communities while KNWD has a total of seven sub-districts, one hundred and seventeen communities. Simple random sampling technique was used to select two sub-districts in each district. Purposive sampling method was used to select health volunteers and health workers for the interviews. First of all, the data collectors contacted the volunteers and solicited information on the number of years they have worked as a health volunteer. Health volunteers who have worked for five years and above were identified and interviewed after informed consent was obtained. Health workers in-charge of volunteer activities at the selected sub-districts were also identified and interviewed. Snowball sampling technique was used to identify dropout volunteers for the interviews. For instance the first dropout volunteer who was identified and interviewed helped the data collectors to identify other volunteers who also left the work. All the participants consented to be part of the study.

### Data collection technique

Two graduates with previous experience in qualitative interview moderation were recruited and trained for data collection. Appointments were made with participants on suitable date and time before the interviews were conducted at home. Each interview took between 30 to 45 minutes. Depending on the expressed preference of the participant, the interviews were conducted in Kassem, Nankani and English. In all, 32 IDIs were conducted in this study. Twenty IDIs were conducted with long serving health volunteers, eight IDIs with dropout health volunteers and four IDIs with health professionals.

### Data processing and analysis

We utilized the principle of saturation for data collection in this study. Data saturation was reached where no new or additional information is being found from the interviews [20]. Data collection, management and analysis were done concurrently. All interviews were audio-recorded and transcribed verbatim. Using QSR Nvivo 10 software, codebooks were developed using a combination of established categories based on the original research questions (examples of basic broad themes included ‘activities of health volunteers’, ‘factors affecting retention of volunteer activities’ etc) and themes that emerged from the data using a Grounded Theory approach [21,22]. The codebooks were flexible and themes were reviewed during data collection. To ensure a fair interpretation of the data, the transcripts were initially coded by two researchers independently. Guided by the objectives of the study, the coding process involved a critical review of each transcript to identify emerging themes from the data. The two coders then met to compare their independently-identified themes. They resolved any divergence by re-reading the relevant sections of the transcripts together, and agreed on the best fit interpretation of the data. The major and sub-themes are discussed below, supported by relevant quotes from the transcripts.

## Results

### Background information of respondents

Participants were between twenty to fifty-four years of age. However the ages of the study participants were grouped into three categories (20–30, 31–40 and 41+ years). Most of them (twenty-two) were between 41 years and above, seven were 31–40 years while only three were between 20–30 years. Most of them (eighteen) were males while fourteen were females. In terms of their level of education, majority (sixteen) of them completed primary/junior high level. Twelve of them had senior high/tertiary education while only four of them never had formal education. In terms of their occupation, seventeen were farmers; six were civil/public servants while nine of them were traders/housewife. Most of them (twenty-five) were married; five were widowed /divorced while only two of them were not married.

### Factors motivating health volunteers into volunteer activities

Most of the dropout and long serving health volunteers reported that it was mainly the desire to help their own community members that attracted them to offer their services voluntarily. Some volunteers mentioned that prestige and recognition as doctor in the community were other factors that motivated them to work as health volunteers. A long serving and dropout health volunteers offered their views this way on the issue:

*“What motivated me to do* *this work is to help my community members. If you say you will not do the work it means you do not want to help your own people. Though, the work has no pay but if you refuse to work, it means you do not have your community people at heart” **(IDI-51 year old long serving female volunteer).***

*“What I can say* *made me to work as health volunteer in this community is to support the health workers to provide health care services to the community people. I wanted my community members to be healthy and that made me to do the work. The other thing is that some of the community people call the health volunteers community doctors and that for me attracted me into the work” (**IDI-42 year old long serving male volunteer**)*

The health workers on the other hand were of the view that the frequent interaction with community health volunteers by formal health workers and the allowances given to health volunteers motivated them to work as health volunteers. As one of the health workers put it:

*“Yes, first of all I think* *it is the recognizing and then also the frequent interaction with them by health staff makes them fell part of the health system. Then also once a while we organize some exercise for them and give them some small money also motivate them to work. But I think the most important thing is the recognizing both at the community level and on the part of the health workers. The money that we give them they normally complain that it is small and that they will stop working”**(IDI-40 year old female health worker)**.*

### Health programs health volunteers are engaged at the community level

All the health volunteers and health workers mentioned various health intervention activities and programs health volunteers were involved in at the community level. They mentioned activities such as health education, assisting in weighing of children, mobilizing mothers for polio and other immunizations, counseling services, bed net distribution, providing first aid services, case identification and reporting, elephantiasis drug distribution and treatment of minor illnesses were carried out by health volunteers. Most of the health volunteers were involved in four or more health activities. Study participants shared their views on the issue:

*“The things I do are many. First* *of all when I wake up, I have to go round to see to it that people are healthy. I give health education on how they will keep their houses clean, how to preserve their food and water from contamination and also on the spread and prevention of CSM. At the same time too, I am with Integrated Management of Childhood Illnesses (IMCI) where they have given us some drugs for first aid. After talking to the mothers if they say complain that a child is having stomach pain, diarrhea or headache then I have to give the first aid. Also, when there is going to be a durbar in the community, I go round to inform the community members to come for the durbar and listen to the health authorities…. I am also involved in polio immunization, elephantiasis drug distribution and I advise sick people to visit the nearest clinic for treatment” **(IDI-47 year old long serving male volunteer).***

*“Well, they are* *involved in almost all the health intervention programs going on in this sub district. For example, they are involved in child welfare clinics, they help the nurses in the outreach programs, they help to mobilize community members anytime there is health activity or when there is weighing, they help take the weight of the children, they do compound visits to provide health education and also to inform mothers on weighing days for them to bring their children for weighing. They also help to mobilize community members whenever there is durbar in the community. The volunteers also identify and provide first aid on simple malaria cases to children less than five years but are supposed to refer the serious cases that they cannot handle to the clinic for treatment” **(IDI-36 year old female health worker)***

### Factors affecting retention and sustainability of health volunteers’ activities

Most of the dropout health volunteers said that the inability to combine the voluntary work with their own private activities made them to stop the voluntary work. They also mentioned the attitude of some community members where their efforts were not being recognized and respected was the reason why they stopped working as health volunteers. They reported the issue of old age as one factor that also made some of the health volunteers to drop out of the work. Two of them said that they had to drop out because they were not provided with the logistical supplies they needed for the work. Dropout volunteers expressed their views this way on the issue:

*Q: Can you please tell* *me the reasons why you left the health voluntary work you were doing in your community?*

*R: “The reason why I left the* *work is because it came a time I could not combine the work with my own activities and that is why I had to stop and not that I did not want to do the work. The other people I know who have also left the work is because of old age” **(IDI-49 years old dropout male volunteer)***

*R: “Hmmm, the* *issue has to do with the things (refers to logistical supplies) you need to do the work. They health workers will not give us bicycles to enable us go round the community and that has been the problem. So for me, the bicycle I was using to do the work got spoiled and they did not want to give me new one and that made me to stop the work” **(IDI 33 year old dropout male volunteer)***

*R: “Sometimes* *it comes from the community; they make some volunteers to stop the work and say after all they are not paid. The community thinks volunteers are being paid and so when the volunteers go on compound visits, some people talk nonsense to them. They tell them, “you are paid to work, if they were not paying you, would you be working?” If they say such things and you are quick tempered person; there will be a fight and the volunteer might stop doing the work. I think these are the reasons why” **(IDI-54 year old dropout female volunteer).***

Majority of the long serving volunteers mentioned lack of incentives and logistical supplies such as raincoats, torch lights, wellington boots and transportation in the form of bicycles to facilitate their movement affected the work. They were of the view that lack of these incentives especially bicycles to facilitate their movement had made some of their colleagues to stop working as health volunteers.

*“This is a voluntary work* *that we are doing to help our own people, but the problem is that they will not give us the things (refers to logistical supplies) we need for the work and that is the problem we have with the nurses. You are supposed to go round the community and tell mothers to come for weighing and sometimes when there is a medicine that the nurses want to give to the people in the community, they call us to help them. In the raining season, if you don’t have raincoat you cannot do the work because of the rain. They will not also give us bicycle to use for the work? I think this is the reason why some of them stopped doing the work” **(IDI-45 year old long serving female volunteer)***

*The truth is that we are not being paid for doing this work*. *Even though*, *we know that the work is voluntary but we are not motivated to work*. *They should provide bicycled to facilitate the work because you cannot be able to walk and do this work effectively without bicycle*. *For me because we don’t have these things to help us to the work has made some of my colleagues to stop doing this work but if you ask them they will not say it*. **(IDI-38 year old long serving male volunteer)**

However, most of the long serving health volunteers mentioned good health and the desire to help their own community members as motivation for them to continue to engage in health intervention activities in their communities.

*Q: What motivated you* *to continue to work as health volunteer in this community?*

*R: For me, it is because of* *the care and the help we are giving to our own people in the community. This is what I will say is the main reason why some of us are still doing this work. I have worked for 15 years now and I would have stopped doing this work because they are not paying me. But what has made me to still be doing this work is that I want my community members to have good health and because of that l have to sacrifice my time to do the work” **(IDI-37 year old long serving male volunteer)***

*R: Well, as I* *said earlier we are doing this work because we want the people especially children to be healthy in this our community. If you say you will not do it because of money, who will come and do it for you? So we have to do it for our children to be healthy. **(IDI-44 year old long serving female volunteer)***

### Mechanisms to retain and sustain community-based health volunteers’ activities sacrifice

Most of the volunteers suggested that incentives in the form of awards and provision of bicycles as means of transport for the work could help to retain health volunteers and also sustain their activities. They also said that provision of logistical supplies such as raincoats, torch lights, wellington boots could enhance the work and also motivate them to continue to serve their own community members. They were of the view that though it was voluntary work, they could be given allowance to motivate them to work. They said that these were mechanisms that could help retain health volunteers engaged in community-based health intervention programs.

*Q: What do you* *think could be done to help retain and sustain the activities of health volunteers in this community?*

*R*: *“In fact they should give us incentives to also encourage us to do the work although it is voluntary but at least something small for us to use and buy soap*, *it is enough for us*. *They should call us every quarter for refresher training and also give hard working volunteers awards to motivate them to work” (***IDI- 49 year old long serving male volunteer)**

*R: “What I can say* *is that they should support volunteers with the necessary things they need to work with especially bicycles, they need the bicycle to go round and mobilize mothers for health program in the community” **(IDI 34 year old dropout male volunteers)***

*R: “If you don’t* *have bicycle, you cannot walk round all the communities to do the work. The bicycle that they gave me since 1995 it is very old and cannot be used again for the work…. For salary, we know that it is voluntary and we know that we are just to help our own people and our blessings will come from God more than even salary but they should encourage us with something small” (**IDI- 50 year old long serving female volunteer)***

Few of the volunteers and health workers mentioned support from community members in their farms could help reduce the workload of volunteers and this would motivate them to continue to assist in providing basic health care services to community members.

*“For the community members, during the raining season, they* *should at least come and help me on my farm for one day so that I will also get time to go round and do the work. They have not been doing that and that is a problem for us” **(IDI- 54 year old long serving male volunteer-KNWD)***

*“as I said, some of* *them are farmers so if the community can set a small farm for the volunteers so that during the rainy season the community members can engage in farming for the volunteer to support him or her. I think that is good and it will encourage them to also work for them” **(IDI-46 year old female health worker).***

*I think that the community members* *need to know that we are not paid for doing the work and sometimes you have to leave your own work in order to go round the community and mobilize mothers for health meetings and other programs. So during the raining season, they should always try to help us in our farms so that we will also have time to do this work for them. The other thing is that they should try and give us small money to buy soap and also give us bicycles to use for the work” **(IDI- 26 year old long serving male volunteer)***

In addition to that, all the health workers who were interviewed mentioned effective supervision and quarterly meetings with volunteers by health managers were mechanisms that could boost the morale of health volunteers to continue to engage in health activities at the community level. They also mentioned the issue of honesty and sincerity in engaging the volunteers in community-based health intervention programs. They said this could help to build trust and robust working relationship between the health system and the health volunteers. They were of the opinion that these could help to retain and sustain health volunteer activities at the community level.

*“I think the best thing is that* *whatever is due them (volunteers) should be given to them….As for the volunteers, you must be frank with them because when they get to know your weakness, you cannot work with them. So they should make sure whatever that is due the volunteers they should give them. Then we should also intensify our supervision and also have frequent meetings with them. When we sit and we even buy pure water to drink they will know that we have them at heart that is why we sit with them. But when we just leave them, we will not supervise them, you won’t go there, you won’t ask of their problems, any time you call them, then you are asking for reports, they will not listen to you” **(IDI-41 year old female health worker).***

## Discussion

The desire to help community members and the sick motivate health volunteers to engage in community-based health programs. Both long serving and dropout health volunteers in this study suggested that good health and the desire to help their own people motivated them into community-based health interventions. In rural communities where people leave and stay together, they are considered as one family. Therefore, love, care and support for each other are paramount. It is demonstrated that the material expectations is not the only means driving people to work as health volunteers but the satisfaction gained from helping their own community members motivates them to work as health volunteers. Though, health volunteers engaged in community-based health activities are not paid for the work they do, the care and love they have for each other motivate them to sacrifice the little time they have to support health workers to provide basic health care services to people in rural communities. There is cohesiveness and interest in helping one another at the community level and this basically entices people to continue to spend part of their time doing health voluntary work. It is not therefore surprising that some of the health volunteers in this study have been engaged in health voluntary work in their various communities for quite a long time. The interest to help their own community members in terms of their health needs is the basic reason why they continue to engage in the health voluntary work. The desire to improve community members’ health needs and to learn about health related issues motivate people into health volunteer activities [11, 15].

In addition, the recognition at the community level and the status attached to being a health volunteer basically motivate people into community health interventions activities [11]. However, very few of the volunteers in this study mentioned recognition as ‘doctors’ and prestige given by community members attracted them into health voluntary work. It is demonstrated that health volunteer activities could be a possible pathway for people to get future employment because of their involvement in health activities and the level of experience they have acquired [16]. Access to free medicine, incentives and knowledge on health issues have been reported as factors attracting people into health voluntary work [2, 11].

The main factors influencing retention of health volunteers and sustainability of their activities in the study area are means of transport, incentives and logistics. Community level factors such as respect for volunteers and community support also affect retention of health volunteer [15, 16]. Community involvement in selecting of volunteers and supporting them in their activities by contributing in-kind payments appear to be critical to community directed health program sustainability. As reported in other studies, our study also suggested that volunteers who do not get the support of other community members in their farming activities are more likely to drop out of their volunteer work [2, 10, 12]. Lack of community support, respect and negative attitude of some community members were mentioned by dropout volunteers in this study as the main reasons why they left the work. It is important for health program managers to educate community members on the concept of voluntarism and the sacrifices health volunteers are making to support health care delivery at the community level. This would help address the perception of community members that volunteers are being paid to do the work.

Earlier studies reported factors such as respect from professional health workers, positive and supportive co-worker relationship, appreciation by patients and family members support directly influence retention of health volunteers [9, 11]. High targets set by health program managers and lack of promotion affect volunteer activities [11]. Inadequate training and lack of effective supervision reduces the interest and enthusiasm of volunteers and this could affect the retention and sustainability of their activities [23]. These were not mentioned in this study as factors influencing retention of health volunteers.

Incentives such as awards to hard working volunteers, means of transport in the form of bicycle, community support and logistical supplies such as raincoats, torch lights and wellington boots are the main mechanisms that could retain health volunteers and also help to sustain their activities [10, 12]. Means of transport, incentives and logistical supplies have been reported in this study as substantial mechanisms to retain and sustain health volunteers’ activities. Evidence showed that remuneration, community recognition and expectation of getting job in future were retention mechanisms to retain and sustain volunteer activities [11]. Monetary incentive was not mentioned as the main factor affecting retention of health volunteers in this study, they were however of the view that though the work is voluntary, they could be given an allowance to enable them buy soap and wash their cloths. Allowances or non-monetary benefits such as bicycles and radios are considered to have positive influence on the family’s willingness to give permission and also support women to serve as health volunteers [12]. This would help to improve volunteer’s status in the community, provide compensation from the time taken from the family and will be an incentive for long-term service.

Positive attitude of community members towards health volunteers can play a significant role in retaining health volunteers. When community members show respect to health volunteers and also appreciate the work they do, this could motivate them to continue to involve in health activities at the community level. Community and local leaders’ recognition and appreciation could help retain volunteers engage in health activities at the community level [10].

## Conclusion

Health volunteers continue to play an important role in community-based health programs. However, certain factors such as means of transport, incentives, logistical supplies, lack of community support and respect affect the retention and sustainability of health volunteer activities. From the views expressed in this study, monetary incentives may not always be a necessary efficient approach in retaining and sustaining volunteer activities. Therefore, it is recommended that community support and respect for health volunteers could help to retain their activities. Also, when volunteers are provided with non-monetary incentives and logistical supplies such as bicycles, raincoats, torch lights, wellington boots and awards to hard working volunteers, it will help retain them and also motivate them to continue to support health care delivery at the community level.

## Supporting information

S1 File IDI guide for health volunteers. (DOC)

S2 File IDI guide for health workers. (DOC)
