# Supplementary material for: Retention and sustainability of community-based health volunteers' activities: A qualitative study in rural Northern Ghana
Source: PLoS One. 2017 Mar 15;12(3):e0174002. doi: 10.1371/journal.pone.0174002 (PMC5352025; doi:10.1371/journal.pone.0174002)
Supplement: S1 File — (DOC) [file pone.0174002.s001.doc]

**IDI GUIDE FOR VOLUNTEERS WHO HAVE WORKED FOR LONG AND THOSE WHO HAVE LEFT**

**Background information**

Age

Gender

Level of education

Occupation

**Activities and attraction**

1. What exactly do you do as health volunteer in this community?
2. What attracted or motivated you to accept to work as health volunteer?
3. What exactly motivated you to work for these number of years as health volunteer
4. Why did you have to stop working as a health volunteer (*for those who are not working again*)

**Selection, training and performance**

1. How were you selected and trained as a health volunteer?
2. What responsibility does the community have toward your work as a volunteer?
3. What does the community do to help you work well
4. What does the community do that affect or affected your work as a volunteer

**Retention and sustainability of volunteers and health interventions**

1. What is the level of sustainability of community health interventions & volunteers’ activities in this community/sub-district?
2. What general factors do you think affect the retention of health volunteer in this community? **Probe**
3. Management level factors (Funding, training, supervision)
4. Community level factors
5. Individual level factors
6. In your opinion, what are the best ways to help sustain health interventions and activities of CBHVs in this community?
7. What is the best ways or mechanisms health managers could use to retain health volunteers?

**Thank you very much for your time!!!**
