# Supplementary material for: Retention and sustainability of community-based health volunteers' activities: A qualitative study in rural Northern Ghana
Source: PLoS One. 2017 Mar 15;12(3):e0174002. doi: 10.1371/journal.pone.0174002 (PMC5352025; doi:10.1371/journal.pone.0174002)
Supplement: S2 File — (DOC) [file pone.0174002.s002.doc]

**IDI GUIDE FOR HEALTH STAFF**

**Background information**

Qualification/ highest level of education

Age

Sex

**Type of health interventions in the sub-district and activities of CBHVs**

1. What are the health intervention programs you have in this district/sub-district? **Probe:** how long have these interventions been going on in this district
2. What role do you play on these health intervention programs in this district/sub-district?

**Probe:** Role on the activities of the CBHVs, how many staff are involved in the health interventions in the district or sub-district?

1. What are the health interventions CBHVs are involved in this district/sub district?
2. What are the activities that the volunteers are expected to carry out?
3. What are the things that you think attract people to accept to work as health volunteers in this district/sub-district? (what motivate them to work as volunteers)

**Selection, training and performance of CBHVs**

1. How were the community volunteers selected? Probe for the processes involved in the selection
2. How were they trained and deployed into the community?
3. What do you have to say regarding the level of supervision of volunteer activities?
4. What would you say concerning the performance of the health volunteers in this district/sub-district? **Probe:** attending meetings, their involvement in immunization activities, health education, identify and treat minor illnesses etc.
5. What general barriers do you think volunteers face when trying to do their job
6. What responsibility does the community have towards the volunteer? **Probe:**
   1. What do they do to help them
   2. What do they do that affect their work

**Funding and sustainability of CBHVs activities**

1. What are the main sources of funding for the health intervention in this district? Probe: any alternative funding sources locally, what do you do to sustain these health interventions when the main funding ends?
2. How are the programs designed and implemented in this district? **Probe:** What is the level of involvement of the community and stakeholders, what is the level of involvement of other health staff in the activities of community health programs in this district?
3. What are the factors in your opinion that affect retention and sustainability of CBHVs activities? Probe:
   1. Management level factors
   2. Community level factors
   3. Individual volunteer factors
4. What do you think is the best way to sustain
   1. Community-based health intervention programs
   2. The activities of CBHV in your district.
5. What are the mechanisms put in place to retain health volunteers in your district?
